# Early detection and better outcomes: molecular approaches in pediatric hematologic malignancies – a review

**DOI:** 10.1097/MS9.0000000000003723

**Published:** 2025-08-11

**Authors:** Emmanuel Ifeanyi Obeagu

**Affiliations:** Department of Biomedical and Laboratory Science, Africa University, Zimbabwe

**Keywords:** early detection, genetic markers, leukemia, molecular diagnostics, pediatric hematology

## Abstract

Pediatric hematologic malignancies, including acute lymphoblastic leukemia (ALL), acute myeloid leukemia (AML), and various lymphomas, remain among the most prevalent childhood cancers globally. While traditional diagnostic methods – such as morphology, cytochemistry, and immunophenotyping – have improved outcomes over recent decades, they often fail to capture the genetic heterogeneity and subtle prognostic nuances critical for personalized care. Recent advances in molecular diagnostics, including polymerase chain reaction (PCR), fluorescence in situ hybridization (FISH), and next-generation sequencing (NGS), have transformed the diagnostic landscape. These tools enable the detection of key genetic alterations, support minimal residual disease (MRD) monitoring, and guide targeted therapeutic interventions. However, the adoption of these technologies in low-resource settings is challenged by infrastructural, economic, and logistical barriers. This review explores the molecular landscape of pediatric hematologic malignancies, assesses current diagnostic applications, and discusses challenges in resource-limited settings. We also propose actionable recommendations for clinicians, researchers, and policymakers, including strengthening cancer registries with molecular data, establishing regional diagnostic hubs, integrating molecular tools into frontline protocols, and fostering international collaborations to bridge global disparities. By aligning molecular innovation with implementation strategies, we can move toward more equitable and effective care for children affected by hematologic cancers.

## Introduction

Pediatric hematologic malignancies are among the most prevalent childhood cancers, with acute lymphoblastic leukemia (ALL) accounting for approximately 75% of all cases. Other common types include acute myeloid leukemia (AML) and various forms of lymphomas such as Hodgkin and non-Hodgkin lymphoma. While cure rates have improved substantially over the past few decades due to better chemotherapy protocols and supportive care, a significant proportion of children still experience relapse or long-term treatment-related complications. In this context, early and accurate diagnosis becomes pivotal – not only to initiate timely therapy but also to tailor treatment intensity based on individual risk factors^[1–3]^. Traditionally, the diagnosis of hematologic malignancies in children has relied on clinical presentation, morphology, cytochemistry, and immunophenotyping. While these methods remain essential, they may not capture the full genetic landscape of the disease. This limitation has spurred a paradigm shift toward molecular diagnostics, which delve into the genetic and epigenetic alterations driving malignancy. These molecular insights not only aid in establishing a more precise diagnosis but also offer critical prognostic information and therapeutic guidance, especially in a pediatric setting where treatment decisions must balance effectiveness with long-term safety^[4–6]^. The evolution of molecular biology and genomic sequencing technologies has opened new frontiers in pediatric oncology. Tools such as polymerase chain reaction (PCR), fluorescence in situ hybridization (FISH), and more recently, next-generation sequencing (NGS), have become integral to hematologic cancer diagnostics. These techniques allow for the identification of translocations (e.g., *t(12;21)* in ALL), gene fusions (e.g., *ETV6-RUNX1*), and mutations (e.g., *FLT3, NPM1*), many of which are linked with prognosis and treatment response^[7–9]^.

**HIGHLIGHTS**

- Molecular diagnostics such as PCR, FISH, and NGS are transforming early detection and risk stratification in pediatric hematologic malignancies.
- Minimal residual disease monitoring enhances prognostic precision and guides individualized treatment decisions.
- Targeted therapies informed by molecular profiling are improving survival outcomes and reducing toxicity in children with leukemia and lymphoma.
- Challenges in implementing molecular tools in low-resource settings include infrastructural and economic constraints, requiring innovative, scalable solutions.
- Strengthening molecular registries, regional diagnostic hubs, and global collaborations is critical for improved pediatric cancer care.

One of the key advantages of molecular diagnostics is the ability to detect minimal residual disease (MRD), a measure of the number of leukemic cells remaining after treatment. MRD serves as a highly sensitive biomarker of treatment response and has been incorporated into most modern pediatric treatment protocols to refine risk stratification. High MRD levels after induction chemotherapy are associated with a higher risk of relapse, guiding clinicians to consider intensified therapy. Conversely, low or undetectable MRD may support treatment de-escalation, reducing toxicity^[10–12]^. Another transformative impact of molecular diagnostics is in the realm of targeted therapy. By identifying actionable mutations and pathways, clinicians can apply precision medicine approaches that were once unimaginable. For example, the identification of Philadelphia chromosome-positive (Ph+) ALL enables the use of tyrosine kinase inhibitors (TKIs) such as imatinib or dasatinib in combination with chemotherapy. Similarly, molecular diagnostics have informed the use of CAR-T cell therapy, which targets specific antigens such as CD19 in relapsed or refractory B-cell ALL^[13–16]^. Beyond leukemia, molecular profiling has shed light on pediatric lymphomas as well. Translocations involving the ALK gene in anaplastic large cell lymphoma or MYC gene in Burkitt lymphoma are now routinely tested, enabling subtype-specific therapeutic strategies. These insights have also improved our understanding of tumor biology, disease progression, and potential resistance mechanisms, which is especially important in pediatric patients where long-term outcomes and late effects are critical considerations^[17–19]^.

Acute myeloid leukemia (AML) accounts for approximately 15% of pediatric hematologic malignancies, while lymphomas – encompassing both Hodgkin and non-Hodgkin types – constitute about 10–12% of cases. Despite advances in treatment, relapse remains a significant concern, particularly in acute lymphoblastic leukemia (ALL), where data from SEER (Surveillance, Epidemiology, and End Results) and COG (Children’s Oncology Group) studies indicate relapse rates of 15–20%, especially in high-risk subtypes. Molecular profiling has uncovered a diverse range of genetic alterations that contribute to disease heterogeneity and treatment resistance. Mutations in genes such as KRAS, TP53, and NOTCH1 have been implicated in leukemogenesis, poor response to therapy, and adverse outcomes. These insights underscore the critical role of genomic diagnostics in refining prognosis and tailoring therapy in pediatric hematologic cancers^[18,19]^.

## Aim

The aim of this review is to explore the critical role of molecular approaches in the early detection, diagnosis, and treatment of pediatric hematologic malignancies.

## Methods

This narrative review was conducted to synthesize current knowledge on molecular approaches in the diagnosis and management of pediatric hematologic malignancies. A comprehensive literature search was performed using the electronic databases PubMed, Scopus, Web of Science, and Google Scholar. The search strategy included combinations of the following keywords: *“pediatric hematologic malignancies,” “molecular diagnostics,” “acute lymphoblastic leukemia,” “acute myeloid leukemia,” “PCR,” “FISH,” “NGS,” “minimal residual disease,”* and *“targeted therapy.”* The review covered peer-reviewed articles published between January 2010 and December 2023, with a focus on studies involving pediatric populations (age <18 years). Inclusion criteria comprised original research articles, systematic reviews, meta-analyses, clinical trials, and relevant guidelines that discussed molecular diagnostic tools and therapeutic implications in pediatric leukemia and lymphoma. Exclusion criteria included case reports, editorials, non-English language publications, and studies lacking specific relevance to pediatric hematology.

Titles and abstracts were screened independently by two reviewers to determine eligibility. Disagreements were resolved through discussion or by a third reviewer. Full-text articles were then reviewed and categorized into thematic domains: (1) molecular alterations, (2) diagnostic applications, (3) risk stratification and MRD, (4) therapeutic implications, (5) challenges in low-resource settings, and (6) future directions. Although this is a narrative review, efforts were made to ensure methodological rigor through a systematic approach to literature identification and thematic synthesis. While not registered in PROSPERO, this review adhered to general principles of the PRISMA framework where applicable.

### Molecular landscape of pediatric hematologic malignancies

The molecular landscape of pediatric hematologic malignancies is an intricate and evolving field that continues to reshape our understanding of disease pathogenesis, prognosis, and treatment responsiveness in children. Unlike adult cancers, which often arise from a buildup of mutations over time due to environmental exposures and aging, pediatric hematologic malignancies are primarily driven by specific genetic aberrations that frequently originate during fetal or early postnatal development. These genetic events often involve chromosomal translocations, gene fusions, point mutations, or copy number variations that disrupt normal hematopoietic processes, setting the stage for malignant transformation^[20,21]^. In acute lymphoblastic leukemia (ALL), the most common childhood cancer, numerous recurrent genetic abnormalities have been identified that not only influence disease biology but also guide clinical decision-making. One of the hallmark features of pediatric ALL is the presence of chromosomal translocations, such as *ETV6-RUNX1* (t(12;21)), which is associated with a favorable prognosis, or *BCR-ABL1* (Philadelphia chromosome), which indicates a high-risk profile but is amenable to targeted therapy with tyrosine kinase inhibitors. Similarly, the *MLL* rearrangements, particularly in infants, portend a more aggressive disease course and resistance to conventional therapy, underscoring the importance of molecular stratification at diagnosis^[22,23]^.

**Figure 1.**
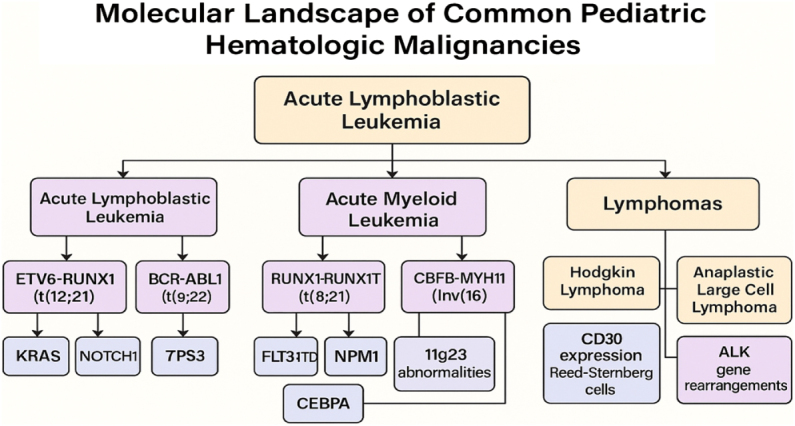
Molecular landscape of common pediatric hematologic malignancies.

In acute myeloid leukemia (AML), which is less common in children than in adults, distinct molecular subtypes have been identified, each with unique clinical implications. For instance, core-binding factor leukemias such as *RUNX1-RUNX1T1* (t(8;21)) and *CBFB-MYH11* (inv(16)) are generally considered to have a good prognosis, especially when molecular remission is achieved. However, mutations in *FLT3, NPM1*, and *CEBPA* genes are increasingly being used to refine risk groups, determine eligibility for hematopoietic stem cell transplantation, and select targeted agents. The discovery of these markers has contributed to more nuanced treatment protocols that go beyond morphological classification alone^[24,25]^. Beyond leukemia, molecular diagnostics are also redefining the understanding of pediatric lymphomas. In Hodgkin lymphoma, genetic studies have revealed alterations in the JAK-STAT and NF-κB pathways, while non-Hodgkin lymphomas, such as Burkitt lymphoma and diffuse large B-cell lymphoma, frequently involve rearrangements of the *MYC, BCL2*, and *BCL6* genes. These genetic profiles help differentiate subtypes and influence the intensity and nature of therapy. For example, ALK-positive anaplastic large cell lymphoma has a better prognosis than ALK-negative cases, prompting the inclusion of ALK inhibitors in ongoing clinical trials for relapsed disease[26]. Importantly, the molecular heterogeneity within these malignancies highlights the need for comprehensive profiling at diagnosis. Advances in next-generation sequencing (NGS) have enabled the simultaneous evaluation of multiple genes and pathways, providing a holistic picture of the mutational landscape in each patient. This technology not only reveals actionable mutations but also uncovers co-occurring genetic alterations that may impact disease progression or therapeutic resistance. Such detailed molecular insights have ushered in the era of precision medicine in pediatric hematology, where treatment decisions are increasingly based on genomic context rather than solely on clinical features (Fig. 1)[27].

Molecular alterations in pediatric hematologic malignancies underpin disease pathogenesis, prognosis, and treatment response. Unlike adult counterparts, pediatric leukemias often harbor age-specific genetic signatures. In acute lymphoblastic leukemia (ALL), recurrent chromosomal translocations such as *ETV6-RUNX1, TCF3-PBX1*, and the *BCR-ABL1* fusion are common and stratify risk levels. Similarly, acute myeloid leukemia (AML) in children features distinctive mutations like *FLT3-ITD, NPM1*, and *CEBPA*, which influence prognosis and therapeutic decisions. These genomic aberrations can co-occur and interact, contributing to disease heterogeneity and resistance mechanisms. Understanding these molecular drivers not only improves diagnostic precision but also guides the integration of targeted therapies such as tyrosine kinase inhibitors or monoclonal antibodies, thereby advancing personalized treatment paradigms in pediatric oncology (Tables 1 and 2)[27].

**Table 1:** Major genetic alterations, their associated pediatric hematologic malignancies, and clinical relevance

Genetic alteration	Associated malignancy	Clinical relevance
ETV6-RUNX1 (t(12;21))	Pediatric ALL	Favorable prognosis; guides treatment de-escalation
Philadelphia chromosome (BCR-ABL1)	Philadelphia-positive ALL	Poor prognosis; indicates need for tyrosine kinase inhibitors (e.g., imatinib)
FLT3-ITD mutation	Pediatric AML	Associated with high relapse risk; guides intensified therapy
NPM1 mutation	Pediatric AML	Generally favorable prognosis; influences treatment choice
KRAS mutations	ALL and AML	Associated with treatment resistance and disease progression
TP53 mutations	Various hematologic malignancies	Poor prognosis; linked with therapy resistance
NOTCH1 mutations	T-cell ALL	Prognostic marker; potential target for novel therapies
ALK rearrangements	Anaplastic large cell lymphoma	Diagnostic marker; targeted by ALK inhibitors
MYC translocations	Burkitt lymphoma	Diagnostic and prognostic; drives aggressive disease
MLL (KMT2A) rearrangements	Infant ALL and AML	Poor prognosis; requires intensive therapy

**Table 2:** Comparative table for molecular techniques (PCR, FISH, NGS) covering sensitivity, specificity, turnaround time, and feasibility

Parameter	PCR (polymerase chain reaction)	FISH (fluorescence in situ hybridization)	NGS (next-generation sequencing)
Sensitivity	High (detects low-level mutations/MRD)	Moderate to high (depends on probe design)	Very high (detects low-frequency variants)
Specificity	High (targeted detection of known mutations)	High (specific chromosomal aberrations)	High (broad detection of known and novel mutations)
Turnaround time	Rapid (hours to 1 day)	Moderate (1–3 days)	Longer (1–2 weeks depending on analysis pipeline)
Cost	Low to moderate	Moderate	High
Infrastructure	Requires basic molecular lab setup	Requires fluorescence microscopy and cytogenetics expertise	Requires advanced sequencing platforms and bioinformatics support
Technical expertise	Moderate	Moderate to high	High
Throughput	Low to moderate (single or few targets)	Moderate (limited by probe multiplexing)	High (simultaneous analysis of multiple genes)
Feasibility in low-resource settings	More feasible with portable/low-cost PCR devices	Limited due to equipment and expertise needs	Challenging due to cost, infrastructure, and expertise

### Diagnostic modalities in molecular hematology

The field of molecular hematology has undergone a transformative evolution, particularly in its diagnostic capabilities, which now serve as the bedrock of personalized medicine in pediatric hematologic malignancies. Early and precise identification of genetic aberrations is essential – not only for confirming the diagnosis but also for stratifying risk, selecting therapies, and monitoring disease response. As the understanding of the molecular underpinnings of blood cancers deepens, so too does the arsenal of diagnostic modalities that clinicians and researchers rely upon to unveil the hidden intricacies of malignant transformation in young patients^[28,29]^. One of the earliest and most widely used tools in molecular hematology is the polymerase chain reaction (PCR). This technique, known for its high sensitivity and specificity, is capable of detecting minute amounts of nucleic acid changes, including fusion transcripts, point mutations, and insertions or deletions. In pediatric leukemia, PCR is particularly useful for identifying hallmark translocations such as *ETV6-RUNX1, BCR-ABL1*, or *MLL* rearrangements. Its ability to detect minimal residual disease (MRD) has made it an indispensable tool in both diagnosis and treatment monitoring, allowing clinicians to make informed decisions about therapy intensification or de-escalation^[30,31]^. Complementing PCR is fluorescence in situ hybridization (FISH), a cytogenetic method that uses fluorescent probes to visualize specific genetic alterations on chromosomes. FISH is especially useful for detecting chromosomal translocations, deletions, and amplifications that may not be apparent through conventional karyotyping. In pediatric hematologic cancers, FISH is frequently used to detect rearrangements in genes such as *MYC, BCL2*, and *ALK*, which carry important prognostic and therapeutic implications. Its relatively rapid turnaround time and compatibility with both interphase and metaphase cells make it a flexible option in clinical settings^[32,33]^.

A more recent and game-changing advancement is next-generation sequencing (NGS). This technology allows for high-throughput, parallel sequencing of entire genomes, exomes, or targeted gene panels, capturing a vast array of mutations in a single assay. In pediatric hematology, NGS has enabled the discovery of novel mutations, gene fusions, and pathways previously unknown to play a role in disease pathogenesis. Importantly, NGS is now being integrated into routine clinical practice for the identification of actionable targets, risk assessment, and monitoring for clonal evolution during or after therapy[34]. Microarray-based comparative genomic hybridization (aCGH) and single nucleotide polymorphism (SNP) arrays also contribute valuable information by identifying copy number alterations and loss of heterozygosity, both of which can affect gene dosage and expression. These techniques are especially useful in complex karyotypes, where multiple structural abnormalities may contribute to disease aggressiveness. Their ability to detect submicroscopic genomic alterations makes them particularly relevant in pediatric cases where traditional cytogenetics may be inconclusive[35]. Another emerging modality is digital droplet PCR (ddPCR), a highly sensitive method that quantifies DNA or RNA molecules with exceptional precision. This technology has shown promise in detecting MRD, especially in settings where quantification is critical to guiding therapy. It also enables detection of rare mutations and provides reproducible results even in low-abundance samples, which is particularly beneficial in pediatric settings where sample volumes are often limited[36]. Epigenetic profiling is gaining traction as well, focusing on DNA methylation patterns, histone modifications, and chromatin accessibility. Aberrant epigenetic regulation plays a significant role in hematologic malignancies, and tools that assess these changes are being explored both diagnostically and as therapeutic guides. For instance, methylation signatures have been used to classify leukemias and lymphomas with ambiguous features, aiding in precise diagnosis and potential treatment targets[37]. Liquid biopsy is an exciting frontier in molecular diagnostics, offering a non-invasive method to analyze circulating tumor DNA (ctDNA) or RNA in peripheral blood. Although still in development for many pediatric applications, liquid biopsy holds great promise for real-time monitoring of tumor burden, mutation profiling, and early detection of relapse. It could revolutionize disease surveillance, particularly for children, by reducing the need for invasive procedures like bone marrow biopsies[37].

The incorporation of molecular diagnostics has transformed the evaluation and management of pediatric hematologic malignancies by enabling early, precise, and risk-adapted diagnosis. Polymerase chain reaction (PCR) is widely used to detect specific gene fusions or point mutations with high sensitivity, while fluorescence in situ hybridization (FISH) facilitates chromosomal rearrangement detection directly within cells. Next-generation sequencing (NGS) offers comprehensive genomic profiling, identifying multiple mutations simultaneously and uncovering novel variants with potential clinical relevance. These tools are critical for subclassifying malignancies, monitoring minimal residual disease (MRD), and tailoring therapeutic regimens. Importantly, molecular diagnostics not only refine initial diagnosis but also support dynamic treatment monitoring and early detection of relapse, thereby contributing significantly to improved survival and reduced treatment-related toxicity in pediatric populations[37].

### Challenges in resource-limited settings

Despite the transformative impact of molecular diagnostics on pediatric hematologic malignancies, their widespread adoption in low-resource settings remains significantly constrained by infrastructural, logistical, and economic limitations. Technologies such as polymerase chain reaction (PCR), fluorescence in situ hybridization (FISH), and next-generation sequencing (NGS) require specialized laboratory infrastructure, uninterrupted power supply, quality control systems, and skilled personnel – all of which are often lacking in many low- and middle-income countries (LMICs). High costs associated with equipment procurement, reagent supply, and maintenance further hinder sustainability. Logistically, delays in specimen transport and limited cold-chain capacity compromise sample integrity and turnaround time, affecting timely diagnosis and therapeutic decisions. Moreover, limited access to bioinformatics support and reference databases challenges the interpretation of complex genomic data, thereby impeding clinical integration[37].

To bridge these gaps, scalable and context-sensitive solutions are urgently needed. The establishment of regional genomic centers – equipped to serve multiple hospitals or countries – may provide a cost-effective hub-and-spoke model to centralize expertise and resources. Public-private partnerships can also play a critical role in technology transfer, subsidized pricing, and training initiatives. Innovations in low-cost, battery-operated portable PCR devices and cartridge-based diagnostic systems offer promising avenues for decentralized testing in rural or underserved areas. Additionally, leveraging cloud-based platforms for bioinformatics analysis and fostering international collaborations can enhance diagnostic accuracy and clinical decision-making. While challenges persist, such strategies hold potential to democratize access to precision diagnostics and ultimately improve pediatric cancer outcomes in resource-limited regions[38].

### Minimal residual disease and risk stratification

In the dynamic field of pediatric hematologic malignancies, the concept of minimal residual disease (MRD) has emerged as a cornerstone of modern risk stratification and treatment tailoring. MRD refers to the small number of cancer cells that persist in the patient after induction therapy and are often undetectable by traditional microscopy or morphology-based assessments. These residual cells, though clinically silent, carry the potential to trigger relapse if not adequately addressed. The ability to detect MRD with precision and reliability has revolutionized how pediatric oncologists approach diagnosis, therapeutic intensity, and prognosis^[38–40]^. Advancements in molecular diagnostics – particularly flow cytometry, polymerase chain reaction (PCR), and next-generation sequencing (NGS) – have enabled clinicians to identify MRD at levels as low as 1 leukemic cell in a background of 10 000 to 1 000 000 normal cells. This level of sensitivity allows for a real-time, granular understanding of a patient’s response to therapy. In pediatric acute lymphoblastic leukemia (ALL), for example, MRD status after induction therapy is one of the most powerful predictors of long-term outcomes. Children who achieve MRD negativity by the end of induction typically have a significantly lower risk of relapse, while those with detectable MRD often require treatment intensification or alternative therapeutic strategies^[41–43]^. Risk stratification, once primarily based on clinical parameters such as age, white blood cell count, and cytogenetic findings, now heavily incorporates MRD results. This shift reflects a more personalized approach to care, in which treatment regimens are adjusted according to a patient’s biological response rather than a rigid classification scheme. For instance, MRD-negative patients may be eligible for reduced-intensity therapy, minimizing the toxic side effects that can have lifelong implications. Conversely, MRD-positive individuals may benefit from early interventions such as hematopoietic stem cell transplantation or enrollment in clinical trials exploring novel agents^[44,45]^.

Importantly, MRD is not a static marker but a dynamic one that can be monitored across different stages of treatment. Serial MRD assessments enable clinicians to detect molecular relapse long before clinical symptoms arise, allowing for preemptive therapeutic adjustments. This has proven particularly valuable in high-risk leukemias and relapsed/refractory disease, where early intervention can significantly improve survival outcomes. The prognostic value of MRD is also being explored in pediatric acute myeloid leukemia (AML) and lymphomas, although standardization in these contexts is still evolving[46]. Moreover, the integration of MRD into risk stratification models is fostering the development of more nuanced, biology-driven treatment algorithms. These models consider not only MRD status but also the molecular and cytogenetic landscape of the disease, creating stratification tiers that better predict outcomes and guide therapy. For example, a child with high-risk cytogenetics who achieves rapid MRD clearance may be reclassified into a more favorable risk group, thereby avoiding overtreatment^[47,48]^. As molecular tools become more sophisticated, efforts are underway to harmonize MRD measurement protocols across institutions and international cooperative groups. Standardization will be crucial to ensuring the reproducibility and comparability of results, especially in the context of multi-center clinical trials. Furthermore, the emergence of NGS-based MRD detection offers unprecedented resolution, capable of identifying rare clones that escape conventional methods. These advances are not only improving prognostication but are also uncovering new biological insights into clonal evolution, treatment resistance, and disease recurrence[49].

### Therapeutic implications and targeted therapies

The rise of molecular diagnostics has ushered in a transformative era in the treatment of pediatric hematologic malignancies, shifting the therapeutic landscape from one-size-fits-all regimens to precision-guided interventions. As clinicians gain deeper insights into the genetic and molecular profiles of childhood leukemias and lymphomas, treatment strategies are increasingly shaped by the underlying biology of the disease. This molecularly informed approach allows for targeted therapies that not only enhance efficacy but also reduce the burden of toxic side effects – a critical consideration in growing children with developing organ systems^[50,51]^. At the heart of these advances lies the identification of oncogenic drivers – mutations, translocations, and signaling aberrations that fuel malignant proliferation. These discoveries have paved the way for targeted therapies, which act on specific molecular pathways with far greater precision than traditional cytotoxic agents. One of the most iconic examples is the use of tyrosine kinase inhibitors (TKIs) in Philadelphia chromosome-positive (Ph+) acute lymphoblastic leukemia (ALL). The presence of the *BCR-ABL1* fusion gene in these patients led to the introduction of imatinib and second-generation TKIs like dasatinib, which have dramatically improved survival rates while minimizing the need for intensive chemotherapy or transplantation^[52,53]^. Beyond TKIs, other molecular targets are being exploited across pediatric hematologic cancers. Mutations in the RAS/MAPK pathway, FLT3, JAK-STAT, and PI3K/AKT/mTOR signaling cascades have all emerged as actionable nodes, inspiring the development of pathway-specific inhibitors. For instance, FLT3 inhibitors are being trialed in high-risk pediatric acute myeloid leukemia (AML) with FLT3-ITD mutations, offering hope for a subgroup that traditionally fares poorly with standard treatment. Similarly, JAK inhibitors are being explored in leukemias driven by cytokine receptor mutations or JAK fusions, highlighting the potential of targeted agents to dismantle disease mechanisms at their root (Table 3)[54].

**Table 3:** Targeted therapies used in pediatric hematologic malignancies, including their molecular targets and indications

Targeted therapy	Molecular target	Pediatric indications	Notes
Tyrosine kinase inhibitors (TKIs)	BCR-ABL1 fusion (Philadelphia chromosome)	Philadelphia chromosome-positive (Ph+) ALL	Imatinib, Dasatinib used alongside chemotherapy
CAR-T cell therapy	CD19 antigen	Relapsed/refractory B-cell ALL	Tisagenlecleucel approved for pediatric ALL
BCL-2 inhibitors	BCL-2 protein	Relapsed/refractory AML and some lymphomas	Venetoclax under clinical investigation in pediatrics
ALK inhibitors	ALK gene fusion	Anaplastic large cell lymphoma (ALK-positive)	Crizotinib approved for ALK+ pediatric lymphomas
FLT3 inhibitors	FLT3 mutations	FLT3-mutated AML	Midostaurin in clinical trials for pediatric AML
CD30-targeted therapy	CD30 antigen	Hodgkin lymphoma, anaplastic large cell lymphoma	Brentuximab vedotin approved for relapsed pediatric HL

**Figure 2.**
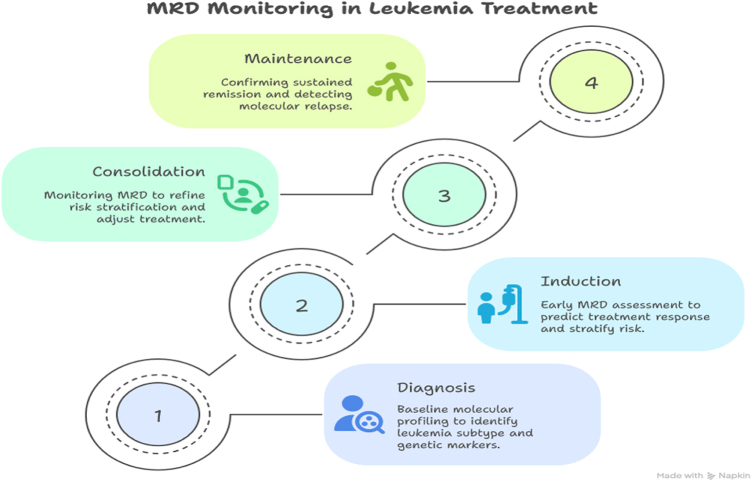
Role of minimal residual disease (MRD) monitoring in pediatric leukemia treatment decision-making.

**Figure 3.**
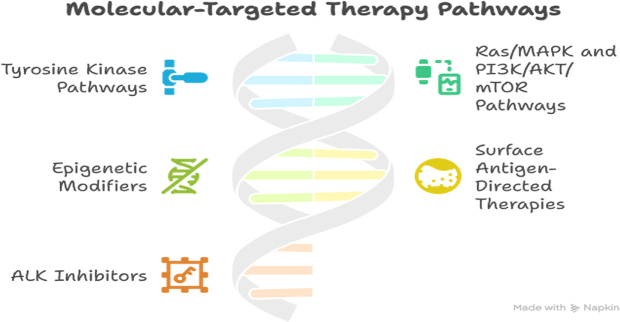
Molecular-targeted therapy pathways in pediatric hematologic cancers.

Monoclonal antibodies and antibody-drug conjugates have also gained momentum as part of the pediatric therapeutic arsenal. Agents such as blinatumomab, a bispecific T-cell engager (BiTE) targeting CD19, and inotuzumab ozogamicin, an anti-CD22 antibody-drug conjugate, have shown promise in relapsed or refractory B-cell ALL. These therapies harness the body’s immune system to selectively eliminate malignant cells, often achieving remission in children who have exhausted conventional options. Their specificity also reduces the collateral damage to healthy tissues, making them suitable for children whose long-term health could be compromised by more toxic treatments[55]. Additionally, the evolution of immunotherapy, particularly chimeric antigen receptor (CAR) T-cell therapy, represents a groundbreaking leap in personalized cancer treatment. Engineered T cells, designed to recognize and attack specific cancer antigens like CD19, have achieved remarkable results in pediatric patients with relapsed or treatment-resistant B-ALL. While challenges such as cytokine release syndrome and neurotoxicity remain, CAR T-cell therapy has opened new avenues for durable remission in cases once considered incurable[56]. The implications of molecularly targeted therapies extend beyond treatment into the realm of therapy de-escalation. For children with favorable genetic markers and excellent response – as indicated by MRD negativity – it may be possible to reduce treatment intensity without compromising cure rates. This strategy not only improves quality of life during therapy but also mitigates long-term complications such as secondary cancers, cardiotoxicity, and fertility impairment, which are particularly concerning in survivors of pediatric cancer^[30,57]^. However, the integration of targeted therapies into frontline treatment remains a careful balancing act. Resistance mechanisms – whether due to clonal evolution, secondary mutations, or pathway bypass – can limit the durability of response. As such, ongoing molecular surveillance and combination therapy strategies are crucial to preempt resistance and sustain remission. Furthermore, access to these novel agents is not uniform across regions, underscoring the need for equitable healthcare infrastructure and cost-effective delivery models (Figs. 2 and 3)[58].

### Challenges and limitations

Despite the remarkable strides in molecular diagnostics and targeted therapies, several challenges and limitations continue to shape the landscape of pediatric hematologic malignancies. While the promise of precision medicine offers great hope, translating molecular discoveries into everyday clinical practice remains a complex endeavor, especially in resource-limited settings and among diverse patient populations[59]. One of the primary challenges lies in accessibility and affordability. Advanced molecular testing platforms such as next-generation sequencing (NGS), quantitative PCR, and flow cytometry require specialized infrastructure, technical expertise, and ongoing quality control – resources that are often scarce in low- and middle-income countries. Consequently, many children with hematologic cancers in these regions are still diagnosed and treated based on outdated protocols, lacking the nuanced insights that modern molecular tools can provide. Even in high-income settings, disparities in access to molecular diagnostics and targeted drugs can lead to inequities in treatment outcomes, underscoring the urgent need for global collaboration and investment in health equity[60]. Moreover, tumor heterogeneity and clonal evolution present significant biological barriers. Pediatric hematologic malignancies, though more genetically stable than their adult counterparts, can still harbor subclones that evolve under therapeutic pressure. These changes can lead to treatment resistance, particularly in the setting of targeted therapies that focus on a single molecular pathway. For instance, while FLT3 inhibitors may show initial success in FLT3-mutated AML, resistant subclones or alternative signaling pathways may emerge, diminishing long-term efficacy. This complexity necessitates continuous molecular monitoring and combination treatment strategies, which may not always be feasible in standard care[61].

Another limitation is the lack of pediatric-specific data. Much of the research and drug development in molecular oncology is initially focused on adult cancers, with pediatric trials often lagging due to smaller patient populations, ethical considerations, and limited financial incentives. As a result, many targeted therapies used in children are based on adult studies, without robust pediatric pharmacokinetic and safety data. This gap can delay regulatory approval and clinical implementation for younger patients, even when a compelling molecular rationale exists[62]. Furthermore, interpretation of molecular data remains a critical bottleneck. While genomic sequencing can uncover a wealth of information, not all identified mutations are clinically actionable. Distinguishing between pathogenic variants and benign polymorphisms requires expert genomic interpretation and functional validation – tasks that demand multidisciplinary collaboration between oncologists, geneticists, and bioinformaticians. The lack of consensus on which molecular findings should guide therapy can lead to variability in clinical decision-making and, at times, overtreatment or missed opportunities for intervention^[59,60]^. Toxicity and long-term effects also pose important concerns. While targeted therapies are often less toxic than conventional chemotherapy, they are not without adverse effects. Tyrosine kinase inhibitors, for example, may cause cardiotoxicity, liver dysfunction, or growth impairment when used long-term in children. Similarly, immunotherapies such as CAR T-cell therapy carry risks of cytokine release syndrome and neurotoxicity, which require intensive monitoring and supportive care. Balancing efficacy with safety – especially in a population that is still developing physiologically – remains an ongoing challenge[61]. The integration of molecular data into clinical workflows requires thoughtful system redesign. From electronic health record compatibility to training healthcare providers on genomic literacy, many institutions are still in the early stages of implementing precision medicine infrastructure. Without streamlined systems for reporting, interpreting, and acting on molecular results, the full potential of these advances may not be realized in time-sensitive clinical settings[62].

### Challenges in low-resource settings and potential solutions

Implementing advanced molecular diagnostics such as next-generation sequencing (NGS), polymerase chain reaction (PCR), and fluorescence in situ hybridization (FISH) in low-resource settings faces significant challenges. These include limited infrastructure, scarcity of trained personnel, unreliable power supply, and the high costs associated with equipment acquisition, maintenance, and consumables. Logistical barriers such as sample transport delays and lack of quality assurance further hinder timely and accurate diagnosis. Additionally, the ethical implications of introducing precision diagnostics in pediatric populations raise concerns around equitable access, informed consent, and potential psychosocial impacts on families when advanced genetic information is revealed. Cost-effectiveness is also a major consideration; while molecular diagnostics can improve outcomes by enabling tailored therapies, their high upfront costs may limit widespread adoption where healthcare budgets are constrained[61].

To bridge these gaps, several scalable solutions can be pursued. Establishing regional molecular diagnostic hubs can centralize resources and expertise, improving access while reducing costs. Public-private partnerships may facilitate funding, technology transfer, and training initiatives. Portable and low-cost PCR platforms offer promising alternatives for basic molecular testing at the point of care. Telemedicine and digital data sharing can support remote interpretation and quality control. Ethical frameworks tailored to local contexts should be developed to guide molecular testing, ensuring patient rights and equity. By combining these strategies, low-resource settings can progressively integrate precision diagnostics, ultimately improving pediatric hematologic malignancy outcomes while maintaining ethical and economic sustainability^[62,63]^.

### Recommendations and future directions

To fully harness the potential of molecular diagnostics in improving outcomes for pediatric hematologic malignancies, a concerted effort is required across clinical practice, research, and policy frameworks. The following actionable recommendations are proposed to guide stakeholders toward more equitable and effective implementation:

1. Strengthening pediatric cancer registries with molecular data

National and regional cancer registries should be expanded to include standardized molecular profiling data. This integration would facilitate better epidemiological tracking, risk stratification, and longitudinal outcome studies. Molecularly annotated registries can also inform public health planning and support clinical trials tailored to local genetic landscapes.

2. Establishing regional molecular diagnostic hubs

To overcome cost and capacity barriers, regional centers of excellence equipped with advanced molecular platforms should be developed. These hubs can serve multiple healthcare facilities, providing centralized testing, quality assurance, and expert interpretation. Such models promote efficient resource utilization and ensure uniform access to high-quality diagnostics.

3. Integrating molecular diagnostics into frontline pediatric hematology protocols

Clinical guidelines for the diagnosis and treatment of pediatric leukemias and lymphomas should embed molecular testing at critical decision points. Incorporating tools like PCR for fusion transcripts or NGS for mutation profiling can support more precise risk-adapted therapies, even in resource-constrained settings where targeted interventions may be limited but prognostic insights are still valuable.

4. Promoting international collaborations for capacity building and research

Global partnerships involving academic institutions, health ministries, and non-governmental organizations can catalyze knowledge transfer, training programs, and joint research initiatives. Such collaborations are crucial for building sustainable local expertise in molecular hematology, enabling participation in multicenter trials and fostering innovation tailored to LMIC contexts.

## Conclusion

The integration of molecular diagnostics into the care of pediatric hematologic malignancies has transformed our understanding of disease biology and ushered in a new era of precision medicine. From early detection and refined classification to risk-adapted therapy and targeted treatment, these molecular insights have significantly improved outcomes for many children facing life-threatening blood cancers. Technologies such as next-generation sequencing, flow cytometry, and real-time PCR have enabled clinicians to identify key genetic mutations, fusion transcripts, and minimal residual disease with greater sensitivity and specificity than ever before. However, while the benefits are clear, the journey toward widespread implementation of molecular medicine in pediatric hematology is far from complete. Numerous challenges persist, including limited access to diagnostic tools in resource-constrained settings, the interpretation of complex genomic data, and the development of effective yet safe targeted therapies for growing children. Equitable access, sustained investment in pediatric-specific research, and collaborative global efforts are essential to close these gaps and deliver on the promise of personalized medicine for all patients.

## Data Availability

Not applicable as this a narrative review.

## Research registration unique identifying number (UIN)

Not applicable as this a narrative review.
